# Combined protein and calcium β-hydroxy-β-methylbutyrate induced gains in leg fat free mass: a double-blinded, placebo-controlled study

**DOI:** 10.1186/s12970-020-0336-1

**Published:** 2020-03-12

**Authors:** Alexander C. Stahn, Martina Anna Maggioni, Hanns-Christian Gunga, Elmarie Terblanche

**Affiliations:** 1 Research Section for Behavioral Regulation and Health, Department of Psychiatry, Perelman School of Medicine at the University of Pennsylvania, 1016 Blockley Hall, 423 Guardian Drive, 19104 Philadelphia, USA; 2 Charité – Universitätsmedizin Berlin, a corporate member of Freie Universität Berlin, Humboldt-Universität zu Berlin, and Berlin Institute of Health, Institute of Physiology, Berlin, 10117 Germany; 3 Department of Biomedical Sciences for Health, Università degli Studi di Milano, via G. Colombo 71, 20133 Milan, Italy; 4 Department of Sport Science, University of Stellenbosch, Private Bag X1, Matieland, 7602 South Africa

**Keywords:** Body composition, HMB, Maximal oxygen uptake, Protein, Strength

## Abstract

**Background:**

The leucine metabolite β-hydroxy-β-methylbutyrate (HMB) is widely used as an ergogenic supplement to increase resistance-training induced gains in fat free mass (FFM) and strength in healthy adults. Recent studies have questioned the effectiveness of HMB, particularly when a high protein diet is habitually consumed. To investigate the additive resistance-training induced effects of HMB and protein in untrained individuals, we conducted a randomized double-blind, placebo-controlled study that compared the effects of combined protein and HMB supplementation to protein supplementation alone on FFM and muscle strength after 12-week resistance training.

**Methods:**

Sixteen healthy men (22 ± 2 yrs) performed a periodized resistance-training program for twelve weeks (four sessions per week). The program comprised two mesocycles, characterized by a linear periodization and non-linear periodization, respectively, and separated by a 1-week tapering period. All participants received 60 g of whey protein on training days and 30 g of whey protein (WP) on non-training days. Participants were randomly assigned to additionally receive 3 g of calcium HMB (WP + HMB) or a placebo (WP + PLA). Body composition and physical fitness were tested before and after the 12-week training program. Whole-body and arm and leg fat free mass (FFM) were assessed by bioimpedance spectroscopy; upper arm and leg fat free cross sectional areas were also quantified using magnetic resonance imaging (MRI); upper and lower body strength were measured by One-repetition maximum (1-RM) bench press and leg press.

**Results:**

Whole-body and segmental FFM increased in both groups (*P* <  0.001). However, gains in leg FFM were higher in WP + HMB vs. WP + PLA (arm FFM: + 6.1% vs. + 9.2%, *P* = 0.2; leg FFM: + 14.2% vs. + 7.0%, *P* <  0.01). No change in fat mass was observed (*P* = 0.59). 1-RM increased in both groups (*P* <  0.001).

**Conclusions:**

Combined protein and HMB supplementation resulted in segmental, but not whole-body increases in FFM compared to protein supplementation alone. These findings could explain some of the controversial effects of HMB reported in previous studies and have practical implications for maximizing training-induced gains in FFM and clinical conditions associated with skeletal muscle deconditioning such as aging, sedentary lifestyles, bed rest and spaceflight.

## Introduction

β-hydroxy-β-methylbutyrate (HMB) is a metabolite of the branched-chain amino acid leucine, which has received increasing interest as an ergogenic aid for training-induced gains in strength and body composition. The mechanisms behind HMB supplementation are related to both anti-catabolic and anabolic effects, enhancing protein synthesis via a mammalian target of rapamycin (mTOR) dependent mechanism, and inhibiting the ubiquitin-proteasome proteolytic pathway responsible for the specific degradation of intracellular proteins [1]. There is also evidence that HMB regulates adipose tissue function including fatty acid oxidation, lipolysis, and adipokine secretion, which may be mediated by mTOR and p-AMP-activated protein kinase α (AMPKα) [2]. Combined HMB-supplementation and resistance training has been reported to positively affect health and physical performance by increasing the fat free mass (FFM) and decreasing fat mass (FM), reducing muscle soreness and improving strength in both previously untrained and trained individuals [3–8]. Increased FFM has also been shown to increase metabolic rate [9], maximal oxygen uptake, and ventilatory thresholds [10, 11]. These adaptations have been attributed to the AMPKα-mTOR pathway and its effect on mitochondrial biogenesis, oxygen consumption and the efficiency of carbohydrate, glycogen and fat metabolism [12]. However, various studies have also questioned the effectiveness of HMB to augment lean mass and performance gains with resistance training in healthy adults [13–17]. Systematic reviews show that about one-third of the studies could not confirm any benefits on strength training-induced effects of HMB on body composition [1, 18, 19]. Some of the conflicting results might be attributed to study designs and methodological issues. In particular, it should be noted that the majority of previous strength training studies on HMB were rather short, lasting eight weeks or less. There is also increasing interest whether HMB supplementation can augment strength training-induced gains in FFM when a high-protein diet is provided [5, 20–23]. Recent studies suggest that protein balance is the critical driver for strength-training induced changes in muscle mass in resistance-trained men [20, 22, 23]. Accordingly, it has been questioned whether HMB supplementation can provide any additional benefits over and above regular protein intake on body composition and performance. To investigate whether a combination of whey protein and calcium HMB exert additive effects on FFM and strength in untrained individuals, we conducted a randomized controlled trial in which healthy young men received a protein supplementation and HMB or protein supplementation and placebo during a periodized 12-week resistance training program.

## Methods

### Experimental design

A randomized, double-blinded, placebo-controlled design was conducted to investigate the effects of a 12-week strength training program and protein supplementation with and without HMB. The study was approved by the Ethics Committee for Human Research at Stellenbosch University and conformed to all standards of human research set out in the declaration of Helsinki. All participants were informed about the purpose, experimental procedures, and risks before giving their verbal and written informed consent.

### Experimental procedures

**Fig. 1:**
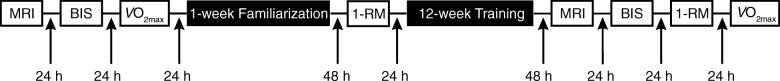
Detailed timeline of training and testing. All tests were separated by 24 h. Tests immediately following completion of familiarization as well as training were performed after a 72 h-resting period to minimize effects related to delayed onset muscle soreness (DOMS) and post-exertion swelling

All participants were provided with a protein and carbohydrate supplement. In addition, the treatment group was supplemented with HMB, whereas the control group was treated with an identically looking placebo (see details below). Group allocation was performed using a stratified randomization procedure to account for the effects of body mass. The total sample was paired based on body mass (i.e., eight pairs), and each participant within each a pair was randomly assigned to either the placebo or the HMB group. After one week of familiarization with the training and testing procedures, all participants performed a full-body strength training program for twelve weeks. Missing more than three training sessions or failure to comply with the supplementation resulted in exclusion from the study. Body composition and physical fitness were tested before and after completion of the strength training program, using the following outcomes. Magnetic resonance imaging (MRI) was performed to determine changes in upper arm and upper leg fat free cross-sectional area (CSA_FFM_). Upper and lower limb, as well whole-body FFM were determined by bioimpedance spectroscopy (BIS). One-repetition maximum (1-RM) bench press and leg press were used to test upper and lower body strength, respectively. Finally, aerobic capacity was assessed by maximal oxygen uptake ($\dot{V}{\mathrm{O}}_{2\mathrm{max}}$). A detailed timeline of testing and training is depicted in Fig. 1. All testing was performed between 9.00 a.m. and 2.00 p.m., while pre and post measurements were performed at approximately the same time for each individual. To ensure normohydration, participants were advised to drink at least 2 to 3 L of water per day during the week preceding the assessments and instructed to refrain from drinking coffee on the days of testing.

### Subjects

Participants were healthy men between 19 and 25 years old, nonsmokers, free of any cardiovascular or metabolic diseases, and orthopedic problems that would limit completion of the study. They had not performed any resistance training exercises or taken any nutritional supplements within the previous six months, were currently not taking medication, ergogenic aids, non-prescription drugs, or any other supplements other than those prescribed by the nutritional protocol during the study. They agreed to abstain from any physical exercise other than that prescribed by the program and agreed to maintain their regular diet throughout the study. Following an interview and a screening of their medical history, a total of 16 healthy men were included in the study. One subject discontinued the intervention because of an accident unrelated to the study. Based on previous studies this sample size was considered sufficient to detect an effect of HMB on FFM [4, 8]. Participants were matched for age, body mass, height, vertical jump height, and strength, and then randomly split into a treatment group who was supplemented with whey protein and HMB (WP + HMB), and a control group who received the same amount of protein and a placebo (WP + PLA).

### Supplementation

Participants consumed 30 g of whey protein powder (EVOX® Advanced Nutrition, Synergy Whey Protein, Johannesburg, South Africa) dissolved in 200–300 ml of tap water immediately before exercise and about one hour after completion of exercise on training days, as well as 30 g of carbohydrate powder (EVOX® Advanced Nutrition, Super Carbo, Johannesburg, South Africa) dissolved in 200–300 ml of tap water immediately after termination of exercise. On non-training days, subjects ingested one serving (30 g) of the whey protein supplement in the mornings only. The additional total energy intake was 1363 kJ (protein: 884 kJ; carbohydrates: 479 kJ) on training days, and 422 kJ on non-training (protein only) days. Details of the supplements are given in the Additional file 1: Table S1. The total amount of essential amino acids, branched-chain amino acids and leucine were about 20.6 g, 9.7 g and 4.4 g for the 60 g servings, and 10.3 g, 4.85 g and 2.2 g for the 30 g servings, respectively. In addition to carbohydrate and protein supplementation, WP + HMB ingested a total of 3 g of HMB daily in the form of capsules, each containing 0.5 g of calcium HMB (EVOX® Advanced Nutrition, HMB, Johannesburg, South Africa). Two capsules were taken three times daily in the mornings, at lunch, and in the evenings. WP + PLA received identical-looking placebo capsules, containing microcrystalline cellulose. The capsules were packed in small sachets, one for each day, each indicating the coded identity of the subject as well as the day of supplementation. The protein and carbohydrate supplementation were supplied in small plastic sachets labeled with each study day. Supplements for one week were handed out at the beginning of each week. Compliance with supplementation was ensured by controlling the empty sachets, assessment of supplement logs by the end of each week, written as well as verbal reminders at each workout session by the training instructors, and personal interviews with each subject regarding the individual progress and program compliance every second week. Dietary habits were not assessed but expected not to change throughout the study as all participants resided on campus and consumed most of their meals from the campus cafeteria.

### Strength exercise training protocol

The strength training protocol consisted of a 12-week, four sessions per week, upper/lower body split routine. Lower body training was performed on Mondays and Thursdays, whereas upper body training was conducted on Tuesdays and Fridays. Subjects rested on Wednesdays and weekends. These days could also be used to make up missed sessions without jeopardizing a minimum of 48 h of rest between two consecutive upper or lower body workouts. The training protocol comprised two mesocycles (weeks 1 to 6 and weeks 8 to 12), which were separated by a 1-week taper phase. During the first mesocycle, training was periodized in a linear, i.e., classical manner by gradually increasing intensity and decreasing volume. The second mesocycle was characterized by a nonlinear, i.e., undulated, periodization by coupling lower (12-RM) with higher (8-RM) intensity workout sessions. In addition, the exercises were slightly varied between two consecutive training days (dumbbell vs. barbell or inclined vs. flat) to ensure varying muscle stress between exercise sessions. A detailed outline of the training protocol is provided in the Additional file 2: Table S2. Exercise intensity was regulated by RM. If the prescribed amount of repetitions could be completed, the weight was increased. All training sessions were performed under the supervision of certified strength training instructors. Each training session was documented in a training log and inspected at the end of each week.

### Strength testing

Employing free-weight bench press and a 45-degree machine leg press, upper and lower body strength were assessed according to standard guidelines [24]. Initially, participants warmed up on a stationary ergometer at low intensity (1 W·kg^− 1^) for 5 min and performed static stretching exercises for an additional 5 min. Subsequently, a light resistance was chosen that easily allowed 5 to 10 repetitions (approximately 50 to 60% of 1-RM). After a brief rest period, this resistance was increased by 5 to 10% for bench press, and 10 to 20% for leg press, and subjects were asked to complete 3 to 5 repetitions. Following a 2-min rest, a conservative, near-maximum load was estimated that allowed subjects to complete 2 to 3 repetitions by further increasing the weight by 5 to 10% for bench press, and 10 to 20% for leg press. After a 2 to 4-min rest, the weight was again increased by 5 to 10% for bench press, and 10 to 20% for leg press, and the individual was instructed to attempt a 1-RM. If the attempt was successful, the weight was increased accordingly, and a further attempt was performed after a 2 to 4-min rest. If the participant failed, a 2 to 4-min rest period was provided and the resistance was reduced by 2.5 to 5% for bench press and 5 to 10% for leg press. Testing was terminated when the participant could not complete one repetition with proper technique. All subjects were given verbal encouragement to facilitate maximal effort contractions. All tests were administered by the same experienced investigator. To avoid learning effects, all participants were accustomed to the testing procedures under the supervision of a certified strength training instructor during the familiarization period.

### Aerobic capacity

$\dot{V}{\mathrm{O}}_{2\mathrm{max}}$ was determined using a maximal graded exercise test on a motorized treadmill using previously described methods [25]. Briefly, subjects performed a 5-min warm-up at a 4% gradient and a self-selected speed. Treadmill grade was then increased by 2% every 2 min until a grade of 10% was reached. If subjects were able to continue exercising after completion of this stage, speed was increased by 0.5 km·h^− 1^ (if the initial selected speed was < 7.0 km·h^− 1^) or by 1.0 km·h^− 1^ (if the initial speed was ≥ 7.0 km·h^− 1^). Oxygen uptake ($\dot{V}{\mathrm{O}}_2$) and carbon dioxide output were determined using an open-circuit breath-by-breath online data acquisition system (Quark b^2^, Cosmed, Rome, Italy). Heart rate was continuously monitored with a heart rate monitor (Polar Vantage, Polar Electro, Kempele, Finland). Post-exercise blood lactate concentration was determined 1 min after termination of exercise using a 5 μL capillary blood sample obtained from the tip of the middle finger (Lactate Pro, Akray, Kyoto, Japan). The highest value of $\dot{V}{\mathrm{O}}_2$ observed over 30 s during the final stages of the test was recorded as $\dot{V}{\mathrm{O}}_{2\mathrm{max}}$. $\dot{V}{\mathrm{O}}_{2\mathrm{max}}$ was assumed, if either a leveling-off of $\dot{V}{\mathrm{O}}_2$ was observed, or if at least two of the following criteria were met: (1) respiratory exchange ratio ≥ 1.15; (2) heart rate no less than 10 beats below age-predicted maximal heart rate; (3) blood lactate concentration > 8.0 mmol·L^− 1^.

### MRI

Fat free cross-sectional areas of the upper arms and upper legs were assessed using a whole-body 1.5 T scanner (MAGNETOM Symphony, Siemens Medical Solutions, Erlangen, Germany). MRI was performed 2 days before the familiarization period and 48 h after the 12-week training protocol (Fig. 1). Transaxial images were obtained by a T1-weighted, spin-echo sequence with a 363 ms repetition time and a 17 ms echo-time. The images consisted of a 50 cm field of view and a 512 × 352 pixel matrix. Subjects were lying in a prone position with their arms stretched overhead and their hands and feet fastened with Velcro straps to prevent rotation. Arms and legs were also slightly elevated using supporters to ensure that all limbs were parallel to the table. After an initial phase of 15 min lying supine to control fluid shifts, the individual turned to the prone position, and sagittal scout images were obtained to determine the most distal part of the humeral and femoral condyles, respectively. Subsequently, 10-mm transversal images were taken 15 cm proximal to the most distal border of the humeral condyles, and 25 cm proximal to the most distal border of the femoral condyles, respectively. Images were transferred to a workstation and analyzed with the National Institutes of Health image analysis software program (Image J1.35, Rasband, W.S. 1997–2006). Fat free cross-sectional areas (CSA_FFM_) of the scans were determined using a semi-automated thresholding technique as follows: at first, thresholds for fat free tissue (including intramuscular vasculature and connective tissue) and adipose tissue (including skin) were selected on the basis of the gray-level histograms of each image. Pixels were then color-coded according to the thresholds, and a semi-transparent copy of fat free tissue was superimposed on the original gray-level image to verify the accuracy of the selected tissue area. Fat free cross-sectional area in each image was then calculated as the sum of the given pixel of the respective tissue area (including bone and intramuscular fat) multiplied by the individual pixel surface area. All images were read and analyzed by a single, trained investigator. Intra-observer differences by experienced investigators are typically < 2% [26].

### Bioelectric impedance spectroscopy

Bioelectric impedance spectroscopy (BIS) was performed with a SEAC SFB3 multifrequency phase-sensitive bioelectrical impedance monitor (Impedimed, Eight Mile Plains, Qld, Australia) via a tetrapolar electrode arrangement following standard procedures that have been previously described in detail [27]. Briefly, current introducing electrodes (2.0 cm × 2.0 cm, Impedimed Inc., Carlsbad, CA, USA) were placed on the dorsal surface of the right hand and foot just below the metacarpal-phalangeal and metatarsal-phalangeal joints, respectively. Whole-body resistance was then determined by positioning two voltage-sensing electrodes (same type as current introducing electrodes) on the dorsal surfaces of the right wrist and ankle midline between the styloid processes of the ulna and radius and midline between the medial and lateral malleoli, respectively. Segmental measurements were obtained by suggestions of Organ et al. [28]. Additional electrodes were placed at the contralateral hand and foot in correspondence to the equivalent positions of the electrodes at the right side of the body. By introducing the current via the right side of the body, resistances of the right arm (i.e., right leg) could be determined by measurements between the voltage-sensing electrodes located at the right and left wrist (i.e., right and left ankle). Resistances of the contralateral side were determined in accordance by introducing the current via the left side of the body. After calibrating the measurement unit according to the guidelines of the manufacturer and lying 10 min supine to allow body fluids to stabilize, a sinusoidal current of 190 μA was applied and impedance and phase angle were recorded at 496 logarithmically spaced frequencies between 5 kHz to 1000 kHz. Infinite resistance (*R*_*∞*_), and extracellular resistance (*R*_E_ = 0 kHz) were obtained by fitting impedance data to the Cole-Cole-equation [29] using the software provided by the manufacturer (v1.5. 2003, Impedimed, Mansfield, Queensland, Australia). Subsequently, whole-body fluid volumes were derived on the basis of the Hanai equation [30], and considering differences in limb and trunk geometry. Specific details of the methodology are provided by Stahn et al. [27, 31]. For the computation of total body water (TBW), the second-generation mixture theory equation was employed [32]. Specific resistivities for intracellular and extracellular water were 273.9 Ω∙cm and 40.5 Ω∙cm, respectively [33, 34]. Bioimpedance measurements can be performed with high within-day and between-day reliability, with an average coefficient of variation for within-day and between-day variability of approximately 1 to 2% and 2 to 3%, respectively, when experienced investigators employ strictly standardized measurement protocols [31]. For the device employed in the present study, *R*_E_ and *R*_*∞*_ have been reported to be within about 3% of theoretical values over a range of resistances typically observed in adults [35]. Body mass was measured barefoot, after voiding, in minimal clothes and on a calibrated electronic digital scale to the nearest 100 g. Standing height was measured barefoot to the nearest 0.1 cm using a stadiometer. FFM was derived from TBW assuming constant mean hydration of FFM of 73.2%. The ratio of extracellular to intracellular spaces was used to assess changes in hydration status (e.g., expansion of extracellular fluid space). Similarly, estimates of FFM were obtained for body segments. However, given the uncertainty of how mixture effects are accounted for with segmental measurements [33, 34], segmental TBW was based on the computation of intracellular and extracellular fluid volumes using the simple resistance-volume relationship of a cylinder [31]. Segment lengths were measured between the respective voltage-sensing electrodes to the nearest 0.1 cm using a flexible steel tape (Lufkin, Cooper Tools, Apex, NC, USA). In line with whole-body measurements, specific resistivities were 273.9 Ω∙cm and 40.5 Ω∙cm for intracellular and extracellular water, respectively.

### Statistical analysis

Descriptive data are reported as means and standard deviations unless stated otherwise. Differences between groups at baseline were examined by independent *t*-tests. Whole-body FM and FFM, strength and aerobic capacity were examined using linear mixed models adjusted for body mass with Time (pre vs. post) and Treatment (WP + HMB vs. WP + PLA) as fixed factors and subject as a random factor. Identical models were applied for segmental (arm and leg) FFM, FM and CSA_FFM_, but additionally including side (left vs. right) as a fixed factor. Covariance matrices were determined by restricted maximum likelihood (REML) estimation. *P*-values were obtained by using Satterthwaite’s approximation for denominator degrees of freedom. Normality and homogeneity were checked by Shapiro–Wilk tests and visual inspections of plots of residuals against fitted values. Within-subject differences for each group were assessed using family-wise Bonferroni-corrected paired *t*-tests (corrected for the number of all outcomes per group). Effect sizes were expressed as standardized mean differences (Cohen’s *d*) and 95% confidence intervals using a bootstrapping procedure [36]. The Type I error level was set to 0.05 (two-sided) for all testing. All statistical analyses and graphical illustrations were carried out using the software package R [37].

## Results

Baseline subject characteristics are provided in Table 1. There were no significant differences between groups for age, height, body mass, and body composition. Results from mixed-model ANOVAs are provided in the Additional file 3: Table S3. Body side did not show any significant interaction with Time or Time by Treatment and Side. To achieve optimal parsimony, the models were reanalyzed for all outcomes after excluding body side as an additional predictor. We did not observe any violations of model assumptions, and normality of residuals was confirmed by Shapiro-Wilks tests (all *P*s >  0.307).

**Table 1:** Participant characteristics at baseline

	WP + PLA (*n* = 7)	WP + HMB (*n* = 8)	*t*	*P*
Age, yrs	21.6 ± 1.0	22.6 ± 1.8	−1.4	0.19
Height, cm	180.6 ± 5.1	181.4 ± 5.2	−0.29	> 0.5
Body mass, kg	80.3 ± 10.3	79.4 ± 9.6	0.16	> 0.5
Fat mass, %	24.5 ± 2.6	24.1 ± 2.3	0.45	> 0.5

Values are means ± SD

### Whole-body FFM and FM

**Fig. 2:**
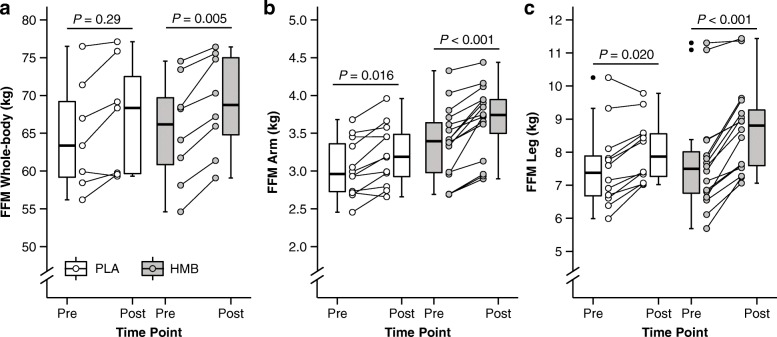
Whole-body and segmental FFM before and after 12-week resistance training with and without HMB supplementation. **a** Whole-body measurements for total body mass and FFM. **b** Arm FFM. **c** Leg FFM. Values are medians (crossbars), interquartile ranges (boxes) and minima and maxima (whiskers). Individual changes are shown by circles and lines. *P*-values indicate within-subject differences for each group using Bonferroni-corrected paired *t* tests. Segmental data include left and side; *n* = 28 (WP + HMB: *n* = 16, WP + PLA: *n* = 12) for arm and leg FFM)

Hydration status as assessed by the ratio of extra- to intracellular water remained unchanged between pre and post (WP + HMB: *P* = 0.16; WP + PLA: *P* = 0.20). Individual changes in whole-body FFM are provided in Fig. 2a. In the WP + PLA group, two subjects showed a strong increase (> 4 kg), two subjects a moderate (2 to 4 kg) and three subjects a small increase or no increase (< 2 kg) in whole-body FFM. In contrast, in the WP + HMB group, only one subject demonstrated a small increase in whole-body FFM, and this change was actually close to 2 kg (i.e., 1.88 kg). Four subjects responded with a moderate, and three subjects with a strong whole-body increase in the WP + HMB group. Whole-body FFM significantly increased from baseline to week 12 in both groups (*P* <  0.001 for Time) (Fig. 2). The average gains in whole-body FFM were more pronounced in WP + HMB, which increased from 65.4 ± 7.1 to 68.8 ± 6.6 kg compared to a change from 64.7 ± 7.4 to 67.0 ± 7.7 kg in WP + PLA (note that Fig. 2 displays medians not means). Simple comparisons for each group showed that this increase was significant for WP + HMB (*P* = 0.005), but not for WP + PLA (*P* = 0.29). The interaction between Treatment and Time was not significant (*P* = 0.26). Whole-body FM remained unchanged in both groups, showing no main or interaction effects (*P* = 0.59 and *P* = 0.56 for Time and Time by Treatment, respectively).

### Segmental FFM

**Fig. 3:**
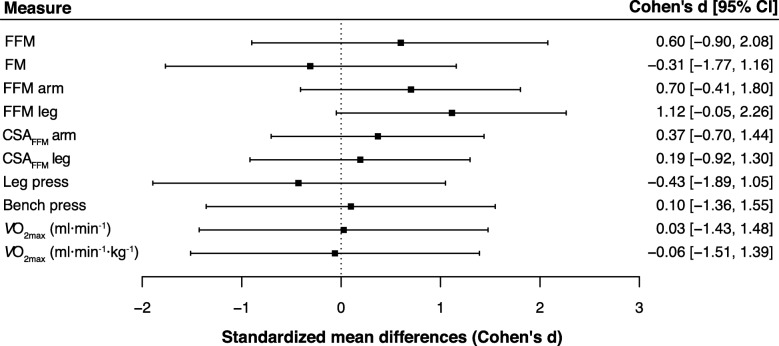
Effect of HMB on body composition, strength, and cardiopulmonary fitness after a 12-week resistance training program. Effect sizes are Cohen’s *d* for differences between change scores. *P*-values are based on *t*-statistics for unpaired comparisons. CI, confidence interval adjusted for multiple comparisons, i.e., 99.5%). CSA_FFM_, FFM cross-sectional area. $\dot{V}{\mathrm{O}}_{2\mathrm{max}}$, maximal oxygen uptake

Changes in arm and leg FFM are shown in Fig. 2b and c (cases include left and right measurements). In WP + PLA, arm FFM increased slightly (< 0.1 kg) in five cases, moderately (0.1 to 0.2 kg) in one case, and strongly in six cases (> 0.2 kg). In the WP + HMB group, all arm FFM gains were moderate to strong (five cases showed moderate increases, and 11 cases demonstrated strong increases). In the WP + PLA group, leg FFM showed small changes (< 0.5 kg) in six cases, moderate increases (0.5 to 1 kg) in five cases, and strong gains (> 1 kg) in one case. By comparison, in the WP + HMB group, three cases showed small changes, six cases moderate gains, and seven cases strong gains. Arm and leg FFM showed a significant effect for Time (*P* <  0.001 for arm and leg FFM, respectively). As shown in Fig. 2, all within-subject comparisons for each group were significant. Changes from baseline to week 12 were similar for arm FFM (WP + PLA + 6.1% vs. WP + HMB + 9.2%), whereas leg FFM indicated larger increases in WP + HMB (WP + PLA: 7.6 ± 1.2 vs. 8.0 ± 1.0 kg, + 7.0%; WP + HMB: 7.7 ± 1.6 vs. 8.7 ± 1.3 kg, + 14.2%). This was confirmed by a significant interaction between Treatment and Time for leg FFM (*P* = 0.007), and very large effect size for the difference in change scores between groups (Fig. 3).

### MRI

Arm and leg CSA_FFM_ were significantly increased in response to the intervention (*P* < 0.001 for arm CSA_FFM_ and leg CSA_FFM_, respectively). The changes from baseline to week 12 were similar between groups (Table 2), with no significant interactions between Time and Treatment (arm CSA_FFM_: *P* = 0.31; leg CSA_FFM_: *P* = 0.77).

**Table 2:** Body composition, strength and maximal oxygen uptake pre- and posttraining

	WP + PLA (*n* = 7)					WP + HMB (*n* = 8)				
	Pre	Post	*t*	*P*	Cohen’s *d*	Pre	Post	*t*	*P*	Cohen’s *d*
Body composition										
Body mass, kg	80.3 ± 10.3	83.2 ± 11.4	− 2.9	0.33	− 1.10 (− 2.5, 0.3)	79.4 ± 9.6	82.8 ± 8.9	− 4.36	0.04	− 1.54 (− 3.1, − 0.03)
FM, kg	15.6 ± 7.0	16.2 ± 7.2	−0.84	> 0.5	− 0.32 (− 1.42, 0.81)	14.0 ± 5.4	14.0 ± 4.8	0.03	> 0.5	0.01 (− 1, 1.02)
FM, %	18.9 ± 7.4	18.9 ± 7.5	0.06	> 0.5	0.02 (− 1.06, 1.10)	17.4 ± 5.8	16.7 ± 5.0	0.87	> 0.5	0.31 (− 0.74, 1.34)
CSA_FFM_ arm, cm^2*^	55 ± 6	59 ± 8	− 4.86	< 0.01	−1.84 (− 3.72, − 0.06)	61 ± 13	67 ± 14	− 5.25	< 0.01	−1.86 (− 3.62, − 0.19)
CSA_FFM_ leg, cm^2**^	160 ± 16	166 ± 16	− 3.72	0.031	−1.41 (− 2.99, 0.14)	161 ± 24	168 ± 25	−5.53	< 0.01	−1.96 (− 3.78, − 0.23)
Maximal strength										
Bench press, kg	54 ± 13	66 ± 16	− 9.4	< 0.001	− 3.55 (− 6.76, − 0.73)	57 ± 12	69 ± 13	− 13.98	< 0.001	− 4.94 (− 8.98, − 1.43)
Leg press, kg	177 ± 35	290 ± 60	−9.58	< 0.001	− 3.62 (− 6.89, − 0.76)	185 ± 52	286 ± 54	− 10.59	< 0.001	−3.74 (− 6.86, − 0.98)
Maximal oxygen uptake										
ml·min^− 1^	4.4 ± 0.5	4.3 ± 0.3	0.74	> 0.5	0.28 (− 0.84, 1.38)	4.3 ± 0.5	4.2 ± 0.5	0.41	> 0.5	0.14 (− 0.88, 1.16)
ml·min^− 1^·kg^− 1^	55.0 ± 6.6	52.5 ± 7.2	1.9	> 0.5	0.72 (− 0.52, 1.93)	54.2 ± 4.1	51.5 ± 6.5	1.4	> 0.5	0.49 (− 0.6, 1.56)

*n = 28 for arm CSA_FFM_ (WP + HMB: *n* = 14, WP + PLA: *n* = 14

**n = 26 for leg CSA_FFM_ (WP + HMB: *n* = 12, WP + PLA: *n* = 14

### Maximal strength

1-RM leg press and 1-RM bench press were significantly increased after the training (*P* < 0.001) (Table 2) with no changes between treatment groups (Time × Treatment: *P* = 0.41 and *P* = 0.85 for 1-RM leg press and 1-RM bench press, respectively).

### Aerobic capacity

There were no changes in absolute $\dot{V}{\mathrm{O}}_{2\mathrm{max}}$ (Time: *P* = 0.49 and Time × Treatment: *P* = 0.96). Relative $\dot{V}{\mathrm{O}}_{2\mathrm{max}}$ was slightly decreased at week 12 compared to baseline (Table 2). The main effect for Time was close to statistical significance for relative $\dot{V}{\mathrm{O}}_{2\mathrm{max}}$ (*P* = 0.051), with no differences for these changes between groups (*P* = 0.91 for Time × Treatment).

## Discussion

We investigated the effects of whey protein supplementation combined with HMB vs. whey protein supplementation and placebo on body composition, strength, and aerobic capacity in healthy, young men after a periodized 12-week resistance training. We found training-induced changes in whole-body and segmental FFM, and maximal strength in both groups. The intake of whey protein plus HMB resulted in larger increases in segmental FFM compared to ingestion of whey protein only.

Our results provide new insights into the discrepancy between previous studies showing that HMB supplementation enhances resistance training-induced gains in hypertrophy and those that could not confirm any additional ergogenic benefits of HMB on body composition. We found increases in whole-body FFM of 2.2 kg in WP + PLA and 3.4 kg in WP + HMB. These results are in agreement with previous studies, reporting gains of about 2 kg in whole-body FFM after twelve weeks of resistance training in young men not receiving any HMB supplementation [4, 6, 8]. In contrast, we could not replicate the large gains in whole-body FFM reported for the HMB supplemented groups in these studies. Kraemer et al. [4], Wilson et al. [8], and Lowery et al. [6] observed gains of about 7 to 8 kg FFM in subjects supplemented with 3 g of HMB per day. Our results are more in line with data recently published by Jakubowski et al. [20], who reported 2.3 kg and 2.6 kg gains in whole-body FFM after 12-weeks of resistance training with protein plus HMB (3 g per day) and protein plus leucine (3 g per day) supplementation, respectively. Such moderate increases in FFM are also supported by a recent meta-analysis, showing that with total protein intake of at least 1.6 g·kg^− 1^·day^− 1^ training-induced gains in FFM can reach a maximum of about 4 kg with an average of about 1.5 kg [38].

Since the difference in gains between WP + HMB and WP + PLA was considerably smaller compared to previous studies [4, 6, 8], we failed to detect a significant treatment effect on whole-body FFM. To verify this, we performed a power analysis using the R package simr. Monte Carlo simulations were used to assess the power for the mixed model investigating the effects of Time and Treatment. One thousand simulations were run to assess power for samples between *n* = 20 and 100. We found that a sample size of *n* = 60 individuals per group would have been needed to detect a significant treatment effect of WP + HMB with > 80% power (α = 0.05, two-sided) if there is one. It is also possible that whole-body BIS might not have been accurate and precise enough to detect any differences in body composition between the groups. Whereas other studies also failed to detect changes in body composition using dual-energy x-ray absorptiometry (DXA) [20], future studies should consider more sophisticated techniques such as DXA, air displacement plethysmography, or magnetic resonance imaging to examine the effects of HMB on whole-body composition.

Segmental FFM seemed to be more sensitive in detecting the effect of HMB. We observed larger effect sizes for gains in segmental FFM compared to gains in whole-body FFM (*d* = 1.12 vs. 0.60 for leg FFM and whole-body FFM, respectively). Increases in leg FFM were significantly larger in WP + HMB (+ 14.2%) compared to WP + PLA (+ 7.0%), whereas no difference in gains was detected for whole-body FFM. We also investigated changes in segmental CSA_FFM_ using MRI measurements. Neither arm nor leg CSA_FFM_ revealed any hypertrophic effect. We attribute this to methodological differences between the approaches for assessing segmental FFM. In line with previous studies, CSA_FFM_ was assessed from a single MRI scan of the arm and leg, respectively. Training-induced changes in muscle volume are not identical along with muscles, and a single slice may not be representative of changes in total limb composition [39]. This is in line with a recent validation study comparing muscle thickness and volume determined by ultrasound and MRI [40]. The authors of the study concluded that a muscle thickness measurement at 50% of vastus lateralis length is a reliable index of muscle volume at a single time point, but poorly tracks changes in muscle volume. There was also no significant correlation between changes in muscle volume and changes in muscle thickness, highlighting the impact of resistance training on regional hypertrophy [40].

In the present study, we also used segmental BIS to determine arm and leg FFM volume. We have previously shown that segmental BIS can provide accurate estimates of arm and leg muscle volume compared to limb volume measurements using MRI [41]. Because segmental measurements for estimating FFM reflect more closely the underlying biophysical model of BIS to whole-body BIS (assumption of isotropic conductor with homogeneous cross-sectional area and consistent proportions specific tissues), we expected segmental measurements to be more accurate and precise than whole-body FFM estimations [27, 28, 31]. The methodological difference between single slice recordings and total limb estimations in tracking changes in segmental hypertrophy may also explain the lack of detecting an ergogenic effect associated with HMB supplementation in two very recent studies, employing similar study designs [20, 22]. However, it should also be noted that the subjects in the present study were untrained men, whereas the afore-mentioned study were performed in resistance-trained men. It is possible that the mTOR and ubiquitin-proteasome proteolytic pathways are already maximized in resistance-trained athletes due to high training loads and the time course of long-term physiological adaptations and explain why muscle protein synthesis may not be augmented in this population. This may be particularly true, when sufficient protein and energy intake is provided [22, 23]. The additional effects of HMB on body composition over and above regular protein intake might therefore only be observed in untrained participants, the elderly population, and bedridden people, but not in resistance-trained athletes [22].

HMB did not affect any of the performance measures that were assessed in the present study. Maximal oxygen uptake remained unchanged. The changes in 1-RM were in a typical range reported in previous 12-week resistance training studies for bench press [3, 8, 20] and leg press [3], but considerably lower than those reported by Kraemer et al. [4] and Lowery et al. [6]. Some of these studies reported no [20] or a slight effect [3] of HMB on 1-RM gains, whereas others found a strong effect [4, 6, 8]. Our findings were in line with a study by Gallagher et al. [42] that examined the effects of HMB, administered in doses of 0 g, 3 g or 6 g per day on FFM and strength during eight weeks of resistance training in 37 untrained men [42]. HMB supplementation resulted in greater increases in FFM compared to a placebo group, but there were no differences in gains of 1-RM between groups. This supports the finding of the present study that longer training programs with combined HMB supplementation could lead to an increase in FFM without any significant changes in 1-RM. It is also possible that the discrepancy between changes in FFM and 1-RM is related to methodologies issues in measuring body composition, i.e., that the increases in FFM are due to an increase of extracellular water and not contractile tissue. Classical bioelectrical impedance analysis performed at 50 kHz is particularly prone to changes between extra- and intracellular because at this frequency the extracellular space dominates the resistance [27, 31]. In the present study we employed bioimpedance spectroscopy that allows to distinguish between intra- and extracellular water based on a biophysical model. Since we did not observe any significant changes in ECW/ICW ratios between pre- and post-testing, suggesting that the changes in FFM are unlikely to be the result of an expansion of extracellular water.

Moreover, whereas Gallagher et al. [42] found no changes in 1-RM, they reported group differences between HMB and placebo supplemented groups in peak isometric and isokinetic torque, stressing the importance of testing specificity for interpreting performance measures of strength. In the present study, care was taken to minimize the effect of non-specific testing, i.e., 1-RM was determined using exercises that were also specifically part of the intervention program (barbell bench press, dumbbell bench press, and leg press). It is possible that testing exercises unrelated to the training program would have revealed larger increases for WP + HMB compared to WP + PLA because of the greater increases observed in segmental FFM, and reducing the effects associated with neurological adaptations on maximal strength for specific exercises.

$Relative\dot{V}{\mathrm{O}}_{2\mathrm{max}}$ decreased slightly as a result of higher body mass, but absolute $\dot{V}{\mathrm{O}}_{2\mathrm{max}}$ was unchanged. This is in contrast to several studies that reported an increase in $\dot{V}{\mathrm{O}}_{2\mathrm{max}}$ after HMB supplementation [10–12, 43]. All of these studies investigated athletes in sports where aerobic capacity and endurance play a key role and/or participants who were exposed to a specific endurance training program during the HMB supplementation. In contrast, in the present study, participants were asked to refrain from any additional exercise other than the strength training program. We suggest that the differences between previously reported effects of HMB on $\dot{V}{\mathrm{O}}_{2\mathrm{max}}$ and our findings could be explained by the lack of intense cardiovascular training in the present study.

The majority of previous studies assessing the effects of HMB on body composition comprised a time frame of eight weeks or less, which may be inadequate to identify the adaptations unique to HMB supplementation [44]. The present study comprised twelve weeks, and according to the authors’ knowledge, this is the longest placebo-controlled study combining HMB and whey protein supplementation to assess strength training-induced effects on body composition. Notably, Jakubowski et al. [20] and Tritto et al. [23] also investigated the effects of HMB supplementation during a 12-week resistance training program. However, the study by Jakubowski et al. [20] was not placebo-controlled, and Tritto et al. [23] did not assess the combined effects of HMB and protein supplementation [20, 23]. Moreover, the training program in the present study was carefully designed and periodized into two mesocycles (weeks 1 to 6 and weeks 8 to 12), which were separated by a 1-week taper phase.

Our findings on whole-body composition confirm recent studies revealing no additional benefit of HMB supplementation over and above regular protein intake on body composition and strength [20, 22, 23]. However, HMB supplementation was associated with significantly greater gains in leg FFM, suggesting that measurements of segmental limb composition are more sensitive to detect smaller changes in FFM. It is possible that a more elaborate timing of the supplementation schedule might have further increased these effects. Exercise stimulates a delayed increase in ubiquitin conjugating activity, which induces a late-phase rise in protein degradation that is critical for muscle adaptation to exercise [45]. Hence, it is possible that the timing of HMB administration in the study was suboptimal. All subjects received protein supplementation immediately before exercise and about 1 h after completion of the exercise, but ingested HMB capsules at rather fixed points in time (in the mornings, at lunch and in the evenings) and not necessarily aligned with the training sessions. However, Wilson et al. [46] showed that HMB consumed 1 hour prior to exercise decreased muscle soreness and reduced the accumulation of lactate dehydrogenase compared to a placebo or when the supplement was administered post-exercise [46]. In line with that, the position stand on HMB as a supplement by the International Society of Sports Nutrition (ISSN) conclude that HMB’s acute effects are likely to be maximized by administering an HMB dose before exercise [1]. However, the authors also recommend that the chronic anabolic effects are optimized by consuming three daily servings of 1 g each.

A critical limitation of the present study is the lack of a non-supplemented control group, which would have provided a better understanding of the resistance-training induced effects per se, irrespective of any protein and HMB supplementation. We also acknowledge that dietary habits were not assessed in the present study, which presents a limitation of the current results because it is possible that the placebo group might have ingested a sub-optimal amount of dietary protein to maximize adaptations to the resistance training program. Finally, it should be noted that the whey protein included 7.3% of leucine. Under normal conditions, approximately 5% (2 to 10%) of leucine is converted to HMB [47]. Based on 60 g and 30 g of protein supplementation on training and non-training days, this corresponds to an additional availability of 0.2 g and 0.1 g (max. 0.4 g and 0.2 g), respectively. We do not expect these doses critically confounded our results by minimizing the effects attributed to the WP + HMB relative to WP + PLA.

## Conclusions

In summary, we conclude that HMB combined with whey protein supplementation can modestly augment training-induced increases in FFM in healthy adults. Notably, we studied untrained individuals and the results cannot be extrapolated to trained athletes. The differentiation of segmental and whole-body measurements could provide increased sensitivity to fully understand the effects of nutritional supplementation on body composition. Our findings are relevant to individuals starting an exercise program and aiming to maximize training-induced gains in muscle mass. This includes clinical conditions associated with muscle wasting or situations of skeletal muscle deconditioning such as aging, immobilization, sedentary lifestyles, bed rest and spaceflight [48]. Further studies are needed to elucidate optimal dosage, administration of HMB and its interactions with different levels of protein intake (i.e., low, moderate, and high protein intake) to identify the most beneficial usage of HMB in these settings.

## Supplementary information

**Additional file 1: Table S1.** Composition of nutritional supplements.

**Additional file 2: Table S2.** Resistance training program.

**Additional file 3: Table S3.** Main and interaction effects of Time and Treatment.

## Data Availability

The datasets generated during and/or analysed during the current study are available from the corresponding author on reasonable request.

## Abbreviations

1-RM: one-repetition maximum
BIS: bioimpedance spectroscopy
CSA_FFM_: fat free cross-sectional area
FFM: fat free mass
FM: fat mass
HMB: β-hydroxy-β-methylbutyrate
MRI: magnetic resonance imaging
PLA: placebo
RM: repetition maximum
TBW: total body water
$$ \dot{V}{\mathrm{O}}_2 $$: oxygen uptake
$\dot{V}{\mathrm{O}}_{2\mathrm{max}}$: maximal oxygen uptake
WP: whey protein
