# Supplementary material for: Combined protein and calcium β-hydroxy-β-methylbutyrate induced gains in leg fat free mass: a double-blinded, placebo-controlled study
Source: J Int Soc Sports Nutr. 2020 Mar 12;17:16. doi: 10.1186/s12970-020-0336-1 (PMC7069016; doi:10.1186/s12970-020-0336-1)
Supplement: Supplementary file 1 — Additional file 1: Table S1. Composition of nutritional supplements. [file 12970_2020_336_MOESM1_ESM.pdf]

Additional file 1. Table that illustrates the composition of nutritional supplements.

**Super Carbo, EVOX® Advanced Nutrition**	**per 100 g**
Energy (kJ)	1597
Macronutrients	
Protein (g)	0
Carbohydrate (g)	95.5
Fat (g)	0
Carbohydrate composition	
Monosaccharides	1.3
Disaccharides	33.9
Trisaccharides	5.9
Higher Saccharides	54.4
Vitamins and Minerals	
Potassium Phosphate	0.1
Magnesium Ascorbate	0.08
Sodium Chloride	0.5
Potassium Citrate	0.15
Zinc Gluconate	0.02

**Synergy Whey Protein, EVOX® Advanced Nutrition**	**per 100 g**
Energy (kJ)	1433
Macronutrients	
Protein (g)	75
Carbohydrate (g)	3.3
Lactose (g)	1
Fat Total (g)	3.3
Fat unsaturated Total (g)	2.4
Medium Chain Triglycerides (g)	1
Mono-unsaturated (g)	0.48
Poly-unsaturated (g)	0.93
Amino acid composition	
Non-essential	
Alanine (mg)	3042
Arginine (mg)	2575
Aspartic acid (mg)	6697
Cystine (mg)	1005
Glutamic acid (mg)	15952
Essential	
Histidine (mg)	1862
Isoleucine (mg)	4174
Leucine (mg)	7329
Lysine (mg)	6131
Methionine (mg)	1950
Phenylalanine (mg)	3273
Threonine (mg)	4061
Tryptophane (mg)	1004
Valine (mg)	4593
Glutamine Peptides (mg)	1000
Glycine (mg)	1532
Proline (mg)	6586
Serine (mg)	4246
Tyrosine (mg)	3327
Minerals	
Calcium (mg)	426
Magnesium (mg)	133
Phosphorous (mg)	278
Potassium (mg)	255
Other nutrients	
Essential Phospholipids (mg)	2000
