# Supplementary material for: Combined protein and calcium β-hydroxy-β-methylbutyrate induced gains in leg fat free mass: a double-blinded, placebo-controlled study
Source: J Int Soc Sports Nutr. 2020 Mar 12;17:16. doi: 10.1186/s12970-020-0336-1 (PMC7069016; doi:10.1186/s12970-020-0336-1)
Supplement: Supplementary file 2 — Additional file 2: Table S2. Resistance training program. [file 12970_2020_336_MOESM2_ESM.pdf]

Additional file 2. Table that illustrates the periodization of the training program.

Exercise	Sets x Repetitions			
	**Week 1-3**		**Week 4-6**	
	MON (LB) TUE (UB)	THU (LB) FRI (UB)	MON (LB) TUE (UB)	THU (LB) FRI (UB)
Lower Body				
Back squat	3 x 12-RM	3 x 12-RM	3 x 10-RM	3 x 10-RM
Leg press	3 x 12-RM	3 x 12-RM	3 x 10-RM	3 x 10-RM
Leg curl	3 x 12-RM	3 x 12-RM	3 x 10-RM	3 x 10-RM
Leg extension	3 x 12-RM	3 x 12-RM	3 x 10-RM	3 x 10-RM
Calf raises seated	3 x 12-RM		3 x 10-RM	
Calf raises standing		3 x 12-RM		3 x 10-RM
Crunch	3 x 25-RM	3 x 25-RM	3 x 25-RM	3 x 25-RM
Upper Body				
Barbell bench press	3 x 12-RM		3 x 10-RM	
Dumbbell bench press		3 x 12-RM		3 x 10-RM
Back lat pull-down	3 x 12-RM	3 x 12-RM	3 x 10-RM	3 x 10-RM
Shoulder press	3 x 12-RM		3 x 10-RM	
Inclined bench press		3 x 12-RM		3 x 10-RM
Seated row	3 x 12-RM	3 x 12-RM	3 x 10-RM	3 x 10-RM
Dips	3 x 12-RM	3 x 12-RM	3 x 10-RM	3 x 10-RM
Triceps pushdown	3 x 12-RM		3 x 10-RM	
Lying triceps extension		3 x 12-RM		3 x 10-RM
Biceps barbell curl	3 x 12-RM	3 x 12-RM	3 x 10-RM	3 x 10-RM
Biceps dumbbell curl seated	3 x 12-RM		3 x 10-RM	
Scott curl		3 x 12-RM		3 x 10-RM

Exercise	Sets x Repetitions			
	**Week 7**		**Week 8-12**	
	MON (LB) TUE (UB)	THU (LB) FRI (UB)	MON (LB) TUE (UB)	THU (LB) FRI (UB)
Lower Body				
Back squat	4 x 5*	4 x 5*		
Leg press	3 x 6*	3 x 6*		
Hack squat			3 x 8-RM	
Lunges			3 x 8-RM	3 x 12-RM
Deadlift			3 x 8-RM	3 x 12-RM
Leg extension†			2 x 15-RM	3 x 12-RM
Leg curl†			2 x 15-RM	3 x 12-RM
Donkey calf raises			3 x 8-RM	3 x 12-RM
Crunch	3 x 25-RM	3 x 25-RM	3 x 25-RM	3 x 25-RM
Upper Body				
Barbell bench press	4 x 5*		3 x 8-RM	
Dumbbell bench press				3 x 12-RM
Back lat pulldown	3 x 6*			
Standing row		3 x 6*	3 x 8-RM	3 x 12-RM
Barbell shoulder press	3 x 6*		3 x 8-RM	
Inclined barbell bench press		4 x 5*		3 x 12-RM
Close grip front lat pull-down			3 x 8-RM	3 x 12-RM
Biceps barbell curl	2 x 6*	2 x 6*	4 x 8-RM	
Biceps Scott curl				3 x 12-RM
Barbell bench press narrow grip			3 x 8-RM	
Triceps extension lying				3 x 12-RM

Training days were split into an upper/lower body split routine. Each body part was exercised twice a week for 12 weeks. Weeks 1 to 6 comprised the first mesocycle of the training program, where training was periodized in linear fashion by gradually increasing intensity and decreasing volume. Following a 1-week taper to avoid overtraining and ensure optimal recovery, a second mesocycle was performed during weeks 8 to 12. During this cycle a nonlinear periodization protocol was employed by varying volume and intensity within each week. Additionally, type and number of exercises varied slightly within each mesocycle to optimize the training stimulus. MON, Mondays; TUE, Tuesdays; THU, Thursdays; FRI, Fridays; LB, lower body; UB, upper body.

* at 70% 5-RM.

† performed as supersets on session 1 (Mondays) of each week. Rest periods were extended from between 1 min and 1.5 min for 12-RM to between 2 min and 3 min for 10-RM and 8-RM, respectively. For crunches rest periods were kept constant at 1 min throughout the study. Repetition velocity was moderate, ranging between 1-2 s for concentric and eccentric muscle actions, respectively.
