# Supplementary material for: Combined protein and calcium β-hydroxy-β-methylbutyrate induced gains in leg fat free mass: a double-blinded, placebo-controlled study
Source: J Int Soc Sports Nutr. 2020 Mar 12;17:16. doi: 10.1186/s12970-020-0336-1 (PMC7069016; doi:10.1186/s12970-020-0336-1)
Supplement: Supplementary file 3 — Additional file 3: Table S3. Main and interaction effects of Time and Treatment. [file 12970_2020_336_MOESM3_ESM.pdf]

Additional file 3. Table that illustrates main and interaction the effects of Time and Treatment.

	df_1	df_2	*f*	*P*
**FFM**				
Time	1	13	38.71	< 0.001
Group	1	13	0.12	0.74
Time x Treatment	1	13	1.41	0.26
**FM%**				
Time	1	13	0.47	0.51
Group	1	13	0.33	0.58
Time x Treatment	1	13	0.37	0.55
**FFM Arm**				
Time	1	40	28.97	< 0.001
Group	1	12	2.83	0.12
Time x Treatment	1	40	1.69	0.2
**FFM Leg**				
Time	1	40	62.24	< 0.001
Group	1	12	0.34	0.57
Time x Treatment	1	40	8.05	0.007
**Arm CSA_{FFM}**				
Time	1	40	56.84	< 0.001
Group	1	12	1.50	0.24
Time x Treatment	1	40	1.07	0.31
**Thigh CSA_{FFM}**				
Time	1	37	14.07	< 0.001
Group	1	11	0.02	0.89
Time x Treatment	1	37	0.08	0.77
**Leg press**				
Time	1	13	203.25	< 0.001
Group	1	13	0.004	0.95
Time x Treatment	1	13	0.71	0.41
**Bench press**				
Time	1	13	256.48	< 0.001
Group	1	13	0.20	0.66
Time x Treatment	1	13	0.04	0.85
**VO_{2max} ml·min^{-1}**				
Time	1	13	0.51	0.49
Group	1	13	0.10	0.75
Time x Treatment	1	13	0.002	0.96
**VO_{2max} ml·min^{-1}·kg^{-1}**				
Time	1	13	4.60	0.051
Group	1	13	0.10	0.76
Time x Treatment	1	13	0.01	0.91

df_1, numerator degrees of freedom df_2; denominator degrees of freedom.
